# Retraction: Improvement of Landfill Leachate Biodegradability with Ultrasonic Process

**DOI:** 10.1371/journal.pone.0096133

**Published:** 2014-04-18

**Authors:** The PLOS ONE Editors

It has been brought to the attention of the *PLOS ONE* editors that this article contains substantial overlap in text and scientific content with the two publications below by the same authors:

E-Journal of Chemistry, Volume 9 (2012), Issue 2, Pages 766-771

http://dx.doi.org/10.1155/2012/820971

 Improvement of Landfill Leachate Biodegradability with Ultrasonic Process

Braz J Chem Eng Vol.29 no.2

http://dx.doi.org/10.1590/S0104-66322012000200003

Use of a sonocatalytic process to improve the biodegradability of landfill leachate

The *PLOS ONE* editors retract this article as we consider that this constitutes redundant publication.

The second author Ali Akbar Roodbari has taken responsibility for the duplication, the other authors were unaware of the different submissions for the manuscript
